# Investigating the relationship between heat-mediated cognitive impairment and antipredator response in a wild bird

**DOI:** 10.1098/rsos.251260

**Published:** 2025-10-08

**Authors:** Camilla Soravia, Benjamin J. Ashton, Sara Piquet-Morón, Alex Thornton, Amanda R. Ridley

**Affiliations:** ^1^ Centre for Evolutionary Biology, School of Biological Sciences, The University of Western Australia, Perth, Western Australia, Australia; ^2^ School of Natural Sciences, University of Chester, Chester, UK; ^3^ College of Science and Engineering, Flinders University, Adelaide, South Australia, Australia; ^4^ Percy FitzPatrick Institute of African Ornithology, University of Cape Town, Rondebosch, Western Cape, South Africa; ^5^ CIBIO, Centro de Investigação em Biodiversidade e Recursos Genéticos, Universidade do Porto, Vairão, Porto District, Portugal; ^6^ Program in Genomics, Biodiversity and Land Planning, BIOPOLIS, Vairão, Porto District, Portugal; ^7^ Centre for Ecology and Conservation, University of Exeter, Penryn, Cornwall, UK

**Keywords:** animal cognition, antipredator behaviour, associative learning, behavioural trade-off, climate change, heat stress, predation risk, vigilance

## Abstract

Increasingly frequent heatwaves require animals to spend more time thermoregulating at the expense of other fitness-related behaviours. Emerging evidence also indicates that high temperatures can impair cognitive function in wild animals. However, whether such heat-mediated cognitive impairment underpins altered behavioural responses during high temperatures remains unclear. We examined the link between naturally occurring high temperatures, cognitive performance and antipredator response in wild southern pied babblers (*Turdoides bicolor*). In a paired experimental design, we performed model predator presentations using a taxidermied common genet (*Genetta genetta*) and a box as the control, and we quantified associative learning performance—a cognitive trait involved in associating predator cues with a threat—for the same individuals under normal and high-temperature conditions. As predicted, individuals showed a stronger antipredator response (combining time spent vigilant, flying and alarming) when presented with the predator compared to the control under normal but not high temperatures. Associative learning performance also declined with increasing air temperatures. However, associative learning performance (whether measured under normal or high temperatures) did not predict the strength of the antipredator response. Our findings provide novel evidence for a reduced antipredator response under high temperatures and suggest that physiological constraints rather than learning impairment might explain this change.

## 1. Introduction

Global warming is increasing both average temperatures and the frequency of heatwaves [1]. Higher temperatures can have both lethal and sublethal impacts on wildlife [2,3]. The most common sublethal impact is represented by the behavioural trade-offs that arise when individuals increase the time spent heat dissipating at the expense of other behaviours, such as foraging and offspring provisioning [2]. Engaging in thermoregulatory behaviours may also limit animals’ ability to respond to predator threats, a behaviour that is crucial for survival (e.g. [4]). However, this potential trade-off between thermoregulation and antipredator behaviour has been rarely quantified in the wild.

When avoiding high temperatures becomes critical to survival, animals may tolerate higher predation risk to avoid lethal heat stress [5–7]. For example, Corregidor-Castro *et al*. [5] reported that lesser kestrel (*Falco naumanni*) chicks were twice as likely to spend time outside unshaded nestboxes (reaching a maximum temperature of 45°C) than shaded ones, even though being outside the nestboxes was likely to result in an increased predation risk. Additionally, a recent playback experiment by Cordonnier *et al*. [8] showed that great tits (*Parus major*) were less likely to approach the speaker and produced fewer mobbing calls under high temperatures in response to broadcasted conspecific mobbing calls, thus engaging less in antipredator behaviour. However, the proximate mechanisms behind altered antipredator behaviour under high temperatures—such as physiological constraints or cognitive impairment—remain to be determined.

Antipredator behaviour may be underpinned by cognition, i.e. the mechanisms by which individuals acquire, process, store and act on information from the environment [9]. Indeed, animals often learn to associate certain cues with a predator threat ([10–12]; but see [13] for an example of innate recognition). Responses to predator threats can be interpreted through the associative learning model of risk sensitivity traditionally applied to describe foraging decisions under uncertainty [14]. In this model, a stimulus (i.e. a predator cue) is associated with a probabilistic outcome (i.e. the likelihood of being predated), and individuals must extract the probability of different outcomes through repeated experiences (i.e. predator encounters) [15]. During a given predator encounter, the individual will sample from the memory representations of the possible outcomes to guide decision-making [15,16]. This process will determine how much time an animal spends engaging in antipredator behaviours versus other behaviours, such as foraging or heat dissipating [17,18]. Therefore, associative learning is a fundamental learning process that underpins the ability of individuals to adjust behaviour adaptively when faced with a predator threat [19,20].

Heat stress can impair cognition in both human [21] and non-human animals [22]. This impairment may result from temperature-dependent changes in neurotransmitter activity and brain biochemistry [23]. Additionally, perceiving an increase in temperature may heighten cognitive load and reduce attentional resources directed to concurrent cognitive tasks [21,24]. Evidence for this has been observed in humans, where heat stress can lead to poorer risk assessment and higher impulsivity [25,26]. Additionally, student exam performance across more than 50 countries declines as the number of hot school days increases, indicating cumulative effects of high temperatures on learning and information retrieval [27]. Similarly, two recent studies in wild birds found that individuals took longer to learn an association under high temperatures [28,29]. A reduced associative learning performance could impair an individual’s ability to accurately assess predation risk and may explain changes in antipredator responses in the heat. However, no study to date has investigated the relationship between antipredator behaviour, individual associative learning performance and environmental temperature in wild animals.

We address this knowledge gap by using southern pied babblers (*Turdoides bicolor*, hereafter ‘pied babblers’) as a study system. Pied babblers are cooperatively breeding passerines endemic to the rapidly warming Kalahari region in southern Africa [30]. We used a paired study design to measure the associative learning performance and antipredator behaviour of the same individuals under normal (<35.5°C) and high temperatures (≥35.5°C, critical threshold identified for an increase in heat offsetting behaviours and decline in foraging efficiency in this species; [31]). To measure antipredator behaviour, we quantified the response of individuals to a taxidermied common genet (*Genetta genetta,* hereafter ‘genet’)—a commonly occurring small mammalian predator that is a threat to adult babblers and is known to access babbler nests at our study site—and compared it to the response to a similarly sized wooden box as a control [32,33]. Given the major role of teaching and social learning during ontogeny in pied babblers [34], including when leading young away from predators [35], we assumed that antipredator responses to genets in the wild are learned through associative learning. Therefore, we predicted that (i) individuals would show a stronger antipredator response when presented with a model predator than a control box, indicating predator recognition; (ii) this antipredator response would be reduced at higher temperatures; (iii) in line with previous findings in this species [29], associative learning performance would be impaired at higher temperatures; and (iv) impaired associative learning performance would be linked to reduced antipredator response under high temperatures.

## 2. Material and methods

### 2.1. Study species and site

Data were collected at the Kuruman River Reserve (KRR; 26°58′ E, 21°49′ S) in the Northern Cape, South Africa, during the austral summers (September–March) of 2022 and 2023. The KRR is located in the semi-arid Kalahari region, where the average summer maximum temperature is 34.5 ± 1.4°C (2005−2020, [36]). Pied babblers are medium-sized passerines (60–90 g) that live in stable social groups, which comprise subordinate helpers and a dominant breeding pair and defend year-round territories of 50−80 hectares [37]. During the study, group sizes ranged from 2 to 7 adults (individuals ≥1 year post-hatching).

The study population is habituated to human presence, enabling direct presentation of cognitive tasks [29]. Birds are individually marked with colour and metal rings for identification. Nestlings are ringed at 11 days post-hatching, and adult immigrants are captured with a walk-in trap for ringing. Age and sex are known for most individuals, with individuals born up to 2019 sexed molecularly via a blood sample (pied babblers are sexually monomorphic; [38]). After 2019, sex was attributed based on sexual behaviours (e.g. courting) and sex-specific chorus calls [39]. Age is known for all individuals ringed as nestlings. Adult immigrants external to the marked population are assumed to be 1 year old upon immigration or 2 years old if they breed in the same summer they immigrate into the study population, as dispersal and breeding are rare before these ages [40,41]. During the breeding season (austral summer), researchers visit each study group weekly to record breeding activity (nest-building, incubation, offspring provisioning and post-fledging care).

### 2.2. Temperature condition

Pied babblers are active from 05:00 to 19:30 during summer, but we collected data between 06:00 and 18:30 to avoid periods when they may be departing from or returning to the roost. Due to logistical constraints, we could not always pair the time of the day for experiments under normal and high temperatures. However, only 5% of the predator presentations and 5% of the cognitive tests were started before 08:00, ensuring that the majority of the data were collected at a time of day in which air temperature could reach the high-temperature threshold of 35.5°C. Additionally, by including time of day as a candidate explanatory term in our statistical analyses, we were able to compare the amount of variance in the antipredator response explained by temperature versus time of day (see §2.6). Cognitive tests and predator presentations under high and normal temperatures were performed in the austral summer (difference in Julian date between temperature conditions: 29 ± 5 s.e. days for associative learning tests and 31 ± 3 s.e. days for predator presentations). Both cognitive testing and predator presentations were always performed in the shade, to avoid any confounding effects due to rapid heating under direct solar radiation [42]. The order of cognitive tests and predator presentations was pseudorandomized for a given individual, ensuring that a model predator presentation never preceded an associative learning test on the same day to avoid potential effects of perceived predation risk on performance on the cognitive task [43]. Prior to cognitive tests and predator presentations, we noted weather conditions (clear or overcast) and measured individual body mass on a top-pan scale (see electronic supplementary material, section S1).

Antipredator response and associative learning performance were quantified for the same individuals under both normal and high temperatures. We randomized whether each individual was first tested in the high- or normal-temperature condition for both associative learning tests and predator presentations, scheduling experiments according to the daily forecast and confirming conditions with field temperature measurements. The high-temperature condition was defined as air temperature equal to or above 35.5°C, which is the threshold at which heat dissipation behaviours increase to the point of causing a decline in foraging efficiency that leads to a net 24 hour body mass loss [31], and the same threshold used in the previous study investigating heat impacts on cognition in this species [29]. We also classified as a high-temperature condition those cases in which air temperature was below 35.5°C (5% of the dataset, air temperature range 34.8–35.3°C), but the bird was displaying heat dissipation behaviours for over 60% of the observation time.

Air temperature was measured in the shade within 5 m of the focal bird using a digital thermometer (RS Pro RS42; resolution: 0.1°C, accuracy: ±(0.5% reading + 1°C)). For predator presentations, which were completed in 10 min at most (see §2.4), air temperature was measured once immediately after. For cognitive tests, air temperature was measured at the start of the test and then every 30 min until the bird reached the learning criterion (see §2.3). If the temperature condition changed during the test (i.e. air temperature reaching 35.5°C or the focal bird displaying heat dissipation behaviours for three consecutive trials when testing under a normal-temperature condition, or *vice versa* under a high-temperature condition) the test was paused and resumed the following day under the same temperature condition [29]. If the temperature condition differed on the second day, we discarded the test and started a new one (using a different colour combination; see §2.3) on a day with the correct temperature condition.

In a previous study, maximum air temperature during testing was the most parsimonious predictor of variation in associative learning performance and was highly correlated with average temperature [29]. Therefore, in the following analyses (§2.6), we used maximum air temperature during cognitive testing. For cognitive tests in which an individual required more than 1 day to reach the learning criterion (tests averaged 1.6 ± 0.1 s.e. days), maximum temperature was the highest air temperature recorded across testing days.

### 2.3. Associative learning test

**Figure 1.**
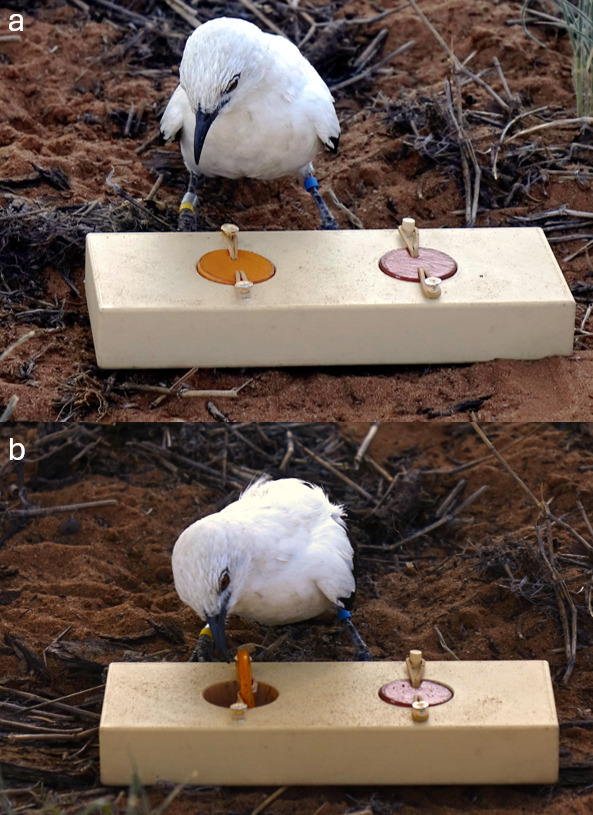
A wild pied babbler interacting with the task used to quantify associative learning performance: (a) the individual approaches the task; (b) the individual opens one of the two wells by pecking at the lid.

To quantify associative learning performance, we used the same protocol employed in previous studies in this population [29] and other studies in wild birds [28,44,45]. The associative learning task consisted of a wooden block (180 × 70 × 30 mm) with two circular wells (30 mm diameter, 20 mm depth) covered by wooden lids secured with elastic bands that allowed the lids to swivel when pecked (see figure 1). Birds were first trained to peck the lids to retrieve a mealworm by using a shaping procedure, a method in which successive approximations of a desired behaviour are reinforced until the full behaviour is reliably expressed. Briefly, the task was presented in four steps: (i) without lids; (ii) with lids on top but not covering the wells; (iii) with lids partially covering the wells; and (iv) with lids fully covering the wells (protocol as described in [29]). The lids used during training were the same shade of yellow, a colour not used during the testing phase. For birds that had already participated in an associative learning test in previous years (7 individuals of 26), the shaping procedure was not repeated. Prior to the first testing day, all of these birds were presented with the training task six times, and we confirmed that each bird reliably pecked and retrieved the mealworm from both lids.

During testing, to minimize the potential influence of colour preference on performance [46], the two lids were painted in different shades of the same colour and the food reward was randomly assigned to one shade (see electronic supplementary material, section S2, for colour details). In the first trial, the focal bird could search both wells to see that only one was baited, but in subsequent trials, the bird was allowed to search only one well. Before the bird could search the second well, the researcher took the wooden task away slowly by initially leaning forward to allow the bird to step back naturally. The task was then kept out of sight for approximately 1 min while the lids were reset and/or the mealworm replaced. Thus, if the bird made the incorrect choice, it could not eat the mealworm for approximately 1 min, introducing a cost for errors.

A bird was considered to have learned the association when it chose the rewarded colour shade in six consecutive trials (binomial test: *p* = 0.016, indicating that the bird was no longer pecking randomly). Associative learning performance was quantified as the number of trials required to reach this learning criterion. If the criterion was not reached within 120 trials, the test was stopped, and the data were excluded from analysis (one instance).

During testing, task orientation was pseudorandomized between trials to prevent individuals from learning an association with the side rather than the colour of the rewarded well. Additionally, we temporarily placed mealworms in both wells prior to the start of testing to control for potential olfactory cues. Finally, to limit the influence of social learning, trials were initiated when the focal individual was temporarily out of sight from other group members. This was possible as pied babblers often forage over 5 m apart [47] and the presence of vegetation obstructs their view of one another. The short duration of each trial (<1 min) typically allowed the subject to complete it before other group members could interfere. If another group member did approach the task during the trial, the trial was stopped and discarded. Additionally, when multiple individuals within a group were tested using the same colour combination, the testing order was recorded to test for potential social learning (*sensu* [44]).

To test each individual under both temperature conditions while controlling for memory effects from the first colour association, we changed the colour of the lids between conditions (see electronic supplementary material, section S2). Finally, to account for potential effects of motivation to interact with the task on performance, we quantified motivation proxies under both temperature conditions (body mass, foraging effort, foraging efficiency, average latency to approach the task and inter-trial interval; see electronic supplementary material, sections S1 and S3).

### 2.4. Predator presentations

**Figure 2.**
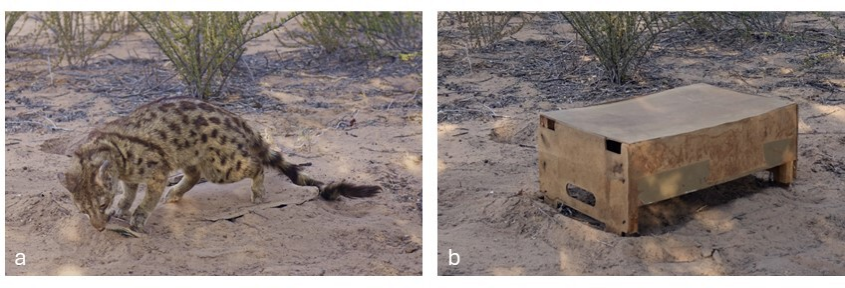
Photos of the taxidermied common genet used as a model predator (a) and the box used as a control novel object (b) in model predator presentations.

Focal individuals were presented with a taxidermied genet and a control box of a neutral colour and similar volume (figure 2) under both normal and high temperatures. The order of the genet and box presentations was randomized within individual and temperature conditions. The box and the genet were presented to the same individual at least 1 h apart and the genet or box was never presented twice in a day to the same group.

Presentations were performed only when the individual was exhibiting normal behaviours and low vigilance and there had not been an alarm call, predator encounter or intergroup interaction in the previous 5 min. Following Dutour *et al*. [48], we placed 2−3 mealworms on the ground to entice the individual to forage in a specific location and ensure a consistent distance of 5 m from the model predator (genet or box). We also made sure that the focal individual was the closest and in line of sight to the model predator. The genet or box was always kept under a cream-coloured blanket, which was cryptic against the sandy background, except during the 1 min presentation time. The researcher first filmed the behaviour of the focal individual for 1 min pre-reveal and then gently pulled away the blanket (by using a string attached to it while sitting approx. 2 m away) and filmed for 1 min post-reveal. During the 1 min post-reveal, the genet was moved by gently pulling a second string connected to its neck once every approximately 5 s, which led to a slight turning of the body to better simulate a real predator [49]. After 1 min, the genet or box was covered with the blanket again. All birds included in this study resumed normal behaviour within 4 min of the reveal. If during the 1 min pre-reveal, other group members approached, the individual flew away or there were other sources of disturbance (alarm calls, intergroup interactions, predators in sight), the filming was stopped before the reveal and the protocol for the predator presentation was started again once normal behaviour resumed. Additionally, if the individual moved out of sight during the 1 min post-reveal during either treatment combination (box or genet normal, box or genet hot), we repeated the full paired experiment, including cognitive testing, in the following field season.

For each predator presentation, we noted the distance from cover and the distance from the model predator of the focal individual at the time of reveal. The latter was noted because while we started by positioning the box or genet 5 m from the individual, the exact distance at the time of the reveal could vary due to the movement of the individual during the 1 min pre-reveal (average distance at the time of reveal 4.0 m ± 0.1 m s.e.). Finally, as group members behind the focal individual could still occasionally notice the predator and emit an alarm, we noted whether other individuals alarmed at the time of reveal.

### 2.5. Behavioural coding

The videos were split into the 1 min pre-reveal and 1 min post-reveal and renamed with a number for blind analysis. Behavioural coding was carried out with the free software *Solomon Coder* (v. 17.03.22, [50]). All behaviours of the focal individual were coded to the nearest 0.2 s (see electronic supplementary material, table S1, for an ethogram), including behaviours typically observed during responses to predators: alarm calling, fleeing and vigilance [51]. We then calculated the proportion of time spent showing any of the behaviours out of the total observation time (1 min minus any time during which the individual was not fully visible because of glare on the camera or partial obstruction by vegetation). If the total observation time was <40 s out of 60 s in the video (either pre- or post-reveal), the video was discarded. Additionally, if during behavioural coding (which allowed for more detailed observation compared to real-time field observations) we recorded that individuals were vigilant for over 30 s during the 1 min pre-reveal, we discarded that presentation (both the 1 min pre- and post-reveal) from further analysis. We also confirmed that the time spent vigilant in a 1 min video was repeatable when coded (i) by the same observer at different times (*R* = 0.93 ± 0.03, 95% confidence interval (CI) = 0.86; 0.96, *n* = 30), and (ii) by two different observers (*R* = 0.89 ± 0.04, 95% CI = 0.78–0.94, *n* = 30) (see electronic supplementary material, section S4).

### 2.6. Statistical analysis

All statistical analyses were performed in *R* software v. 4.4.2 [52]. First, we checked whether the proportion of time spent vigilant pre-reveal differed between treatments (genet and box) by using a Wilcoxon–Mann–Whitney test. Second, we checked whether there was a significant behavioural change before and after the reveal. To test this, we used a generalized linear mixed model (GLMM) fitted with the package *glmmTMB* [53], and we set the difference in the proportion of time spent vigilant between the 1 min post- and pre-reveal as a response variable, predator treatment as a fixed term and individual and group identity as random terms. Subsequently, we focused on the behaviour of the focal individual in the 1 min post-reveal. Following recent studies on antipredator behaviour [54,55], we performed an unrotated principal component analysis using the package *FactomineR* [56] including three behaviours: proportion of time spent flying, alarming and vigilant. These behaviours all loaded positively onto PC1 (proportion of time spent vigilant: 0.64; alarming: 0.68; and flying: 0.72), which accounted for 46.6% of the total variance and had an eigenvalue of 1.4 (see electronic supplementary material, table S2). Therefore, we decided to use individual coordinates along PC1 as a measure of the overall strength of the antipredator response (hereafter ‘antipredator response’).

In the following analyses, we fitted sets of GLMMs where each model contained a single candidate explanatory term as a predictor; additive models and models with interactions between candidate explanatory terms were tested where biologically relevant (see below). Fitted models were compared to each other and to an intercept-only model based on Akaike’s information criterion corrected (AICc) for small sample sizes. The model with the lowest AICc and those within 2 ∆AICc of this model were included in the top model set and were considered to explain variation in the response variable, provided that the 95% CIs of their predictors did not intersect zero [57]. We checked model residuals for normality, overdispersion and outliers with the package *DHARMa* [58].

Before analysing the relationship between antipredator response and cognition, we first tested (i) whether individual attributes or contextual factors affected the antipredator response, and (ii) whether associative learning performance was influenced by temperature, individual attributes, or non-cognitive confounding variables.

For (i), we used a set of linear mixed models with antipredator response (PC1) as the response variable, individual identity and group identity as random terms, and a normal error distribution. The candidate explanatory terms tested were as follows: body mass, time of day, weather (clear/overcast), distance from cover, distance from the predator, presentation number (the cumulative count of box or genet presentations to a group, estimating the maximum exposure per individual to test for habituation), time between presentations (two categories: within 7 days versus over 7 days between presentations of the same predator treatment in the same group), breeding (yes/no, where yes comprises all stages between incubation and the fledglings reaching nutritional independence), individual age, sex, group size (number of adults) and whether other individuals alarmed at the time of reveal (yes/no). We also tested additive and interactive models including predator treatment (genet/box) and one of the covariates above.

For (ii), we used GLMMs with the number of trials to learning criterion as the response variable and individual identity as a random term. Group identity was not included as a random term because it resulted in convergence issues for some models, and it explained slightly less variance (1.50 × 10^–9^) than individual identity (1.55 × 10^–9^). We used a negative binomial distribution with a logarithmic link function to control for overdispersion. The explanatory terms tested were as follows: age, sex, group size, maximum air temperature during testing, time of day, weather (clear/overcast), average latency to approach the task, average inter-trial interval, testing order within a group, rewarded colour shade (light/dark), Julian date (where day 1 = September 1), body mass, individual foraging efficiency and foraging effort. For individuals who were tested more than once under either normal or high temperatures during the study years, only the first replicate per temperature condition was included.

Finally, we used a model set to test our main hypothesis on the relationship between temperature, antipredator behaviour and learning performance. We used linear mixed models with the individual values of PC1 (where PC1 describes the combined antipredator behaviours of an individual) as a response variable and individual and group identity as random terms. The candidate explanatory terms tested were as follows: predator treatment (genet/box), air temperature at the time of the presentation, distance from the predator, associative learning performance measured under normal-temperature conditions (hereafter ‘AL normal’) and associative learning performance matched for temperature condition (i.e. measured under the same temperature condition as the predator presentation; hereafter ‘AL matched’). We also included pairwise additive models and models testing the interaction between predator treatment and the other candidate explanatory terms. Given that our measure of antipredator response (PC1) combined three behaviours (vigilance, alarming, flying), which are likely to differ in terms of energetic cost [59], to aid in the interpretation of the results, we performed a final post hoc analysis by refitting the best model with each of the behavioural variables separately instead of PC1 (see electronic supplementary material, section S6).

## 3. Results

### 3.1. Measuring antipredator response

The final dataset comprised 96 1 min videos pre-reveal and 100 1 min videos post-reveal from 26 individuals from 10 babbler groups. This included 29 box and 28 genet presentations at normal temperatures and 22 box and 21 genet presentations at high temperatures. There was no significant difference in the proportion of time spent vigilant pre-reveal between the genet and the box (Wilcoxon–Mann–Whitney test: Z = 0.09, *p* = 0.93). Post-reveal, there was a steeper increase in the time spent vigilant in response to the genet than the box (predator treatment (box as a reference level): coefficient estimate ± s.e. = 0.22 ± 0.05; 95% CI = 0.12–0.32, *n* = 96 pairs of observations pre- and post-reveal). This supports our prediction that individuals recognize the genet as a potential predator and therefore exhibit an antipredator response.

When exploring factors affecting the overall antipredator response, we found that, within predator treatment (genet or box), individuals responded more strongly when they were closer at the time of reveal (distance predator: coefficient estimate ± s.e. = −0.35 ± 0.11, 95% CI = −0.56 to −0.13, predator treatment (box as a reference level): coefficient estimate ± s.e. = 0.87 ± 0.21, 95% CI = 0.46–1.27, no significant interaction between distance and predator treatment; see electronic supplementary material, table S3).

### 3.2. The effect of temperature on associative learning performance

Out of the 26 individuals presented with the predator and box, 25 completed the associative learning task (one individual did not interact with the task at all and was therefore excluded). Of these, 19 individuals completed the task under both temperature conditions, and 6 only under normal temperatures (total number of tests completed = 44). Of the six individuals who did not complete the test under hot conditions, one reached 120 trials without meeting the learning criterion, while the tests for the remaining five could not be completed due to a lack of sufficient days with high temperatures.

Learning criterion in the associative learning task was reached in an average of 43.9 ± 6.1 s.e. trials under normal temperatures and an average of 46.5 ± 7.6 s.e. trials under high temperatures. As predicted, there was a small but significant effect of temperature on associative learning performance, with individuals taking more trials to reach the learning criterion as maximum air temperature during cognitive testing increased (electronic supplementary material, table S4, figure S1). None of the other individual attributes and non-cognitive confounding factors predicted variation in associative learning performance (electronic supplementary material, table S4). The effect of temperature on associative learning performance remained when including the test in which the learning criterion was not reached and assigning it the maximum score of 120 (temperature: coefficient estimate ± s.e. = 0.26 ± 0.11, 95% CI = 0.04–0.48, ∆AICc compared to the intercept-only model = 2.69, *n* = 45).

### 3.3. The relationship between temperature, cognitive performance and the antipredator response

**Figure 3.**
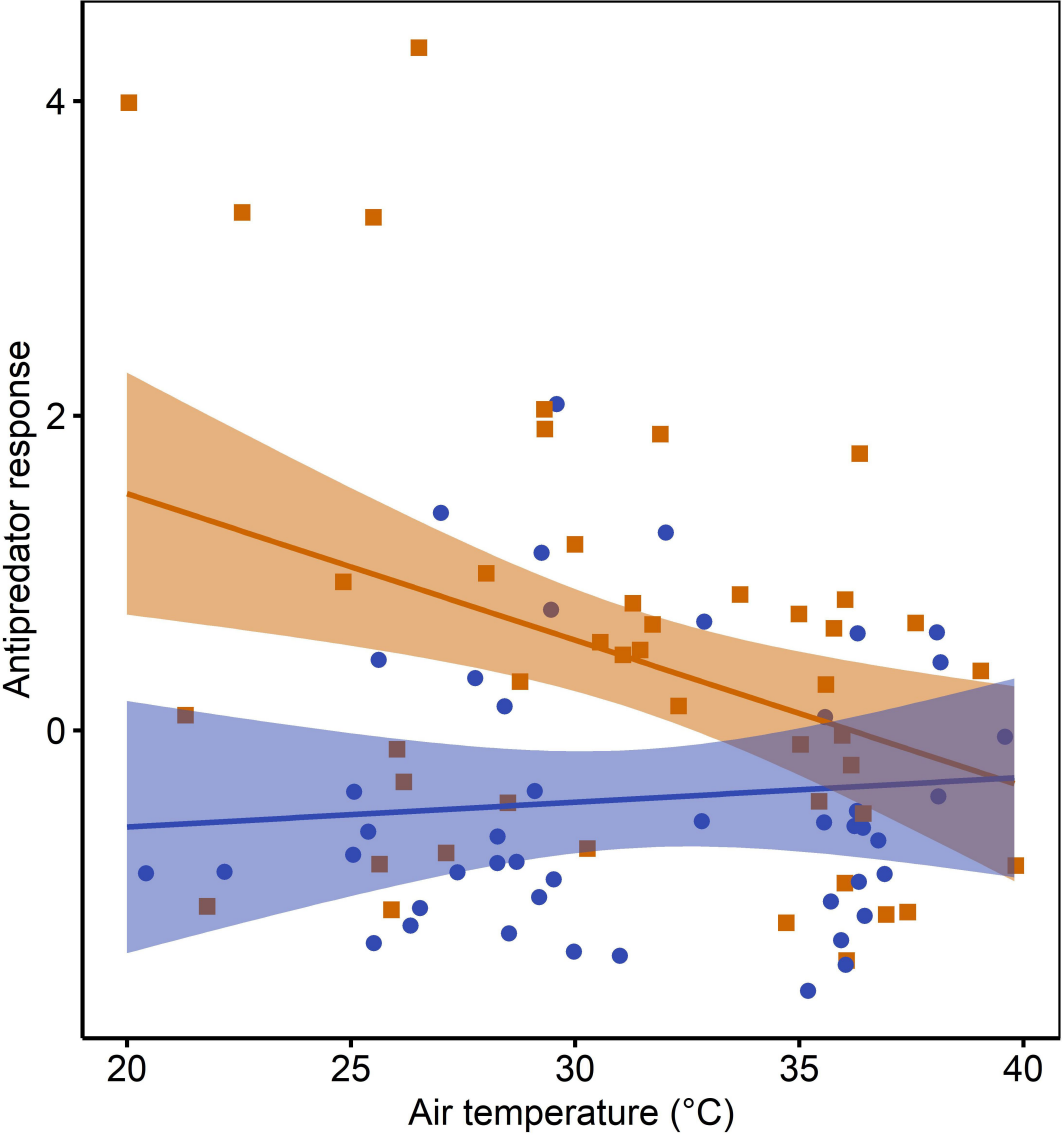
The relationship between the antipredator response (combining time spent flying, alarming and vigilant) and air temperature by predator treatment (genet: orange line and squares, box: blue line and circles) in 25 pied babblers. Points are raw data and have been jittered to reduce overlap. The solid lines and the shaded areas represent the linear regression lines and 95% CI, respectively.

When controlling for predator distance, the best model to explain variation in the antipredator response included the interaction between air temperature during the presentation and predator treatment (table 1). Individuals responded more strongly to the genet than the box at lower air temperatures, but the response to the genet declined with increasing air temperatures (temperature effect for genet presentations: coefficient estimate ± s.e. = −0.10 ± 0.03, 95% CI = −0.16 to −0.04, temperature effect for box presentations: coefficient estimate ± s.e. = 0.01 ± 0.03, 95% CI = −0.05 to 0.07, figure 3). There was no significant difference in the response to the genet and the box at temperatures ≥35.5°C (the previous model accounting for predator distance was refitted separately by temperature condition: 95% CI = 0.81–1.80, *n* = 55, for the effect of predator treatment under normal temperatures, 95% CI = −0.06 to 0.90, *n* = 36, for the effect of predator treatment under high temperatures). These results support our prediction that antipredator response would be reduced in the heat. However, we found no evidence for a link between impaired associative learning and reduced antipredator response. Associative learning performance, measured either under normal temperature (AL normal) or under the same temperature condition as the predator presentation (AL matched), did not predict variation in the antipredator response (electronic supplementary material, table S5).

**Table 1.** Top model set for the terms affecting the antipredator response in wild pied babblers. All models included group and individual ID as random terms. AICc and ΔAICc are provided for models within 2 ΔAICc of the top model and with predictors whose 95% confidence intervals (CI) do not intersect zero. *n* = 91 presentations from 25 individuals in 10 groups. See electronic supplementary material, table S5, for full model selection outputs.

top model set	ΔAICc	weight	k
distance predator + T_air_ × predator treatment	0.00	0.82	8
*Intercept only*	21.91	0.00	4
**effect size of explanatory terms**	estimate	s.e.	95% CI
distance predator	−0.31	0.12	−0.53 to −0.08
predator (genet)	0.88	0.20	0.48 to 1.28
air temperature	0.05	0.15	−0.25 to 0.35
predator (genet) × air temperature	−0.56	0.21	−0.97 to −0.15

## 4. Discussion

Emerging evidence indicates that rising temperatures can alter the antipredator response [7,8] and can negatively affect associative learning performance in wild birds [28,29]. Associative learning is a fundamental cognitive process that allows individuals to associate predator cues with a threat and thus assess predation risk [15,19]. Here, we tested whether impaired associative learning performance underpins the reduced antipredator response observed under high temperatures in wild birds. To this aim, we measured responses to a predator and associative learning performance under normal and high temperatures in the same individuals. As predicted, we found that individuals took longer to learn an association as air temperatures increased. Additionally, individuals responded more strongly to the predator than to the control under normal temperatures but showed a reduced response to the predator as air temperatures increased, resulting in no difference in response between the predator and the control at air temperatures equal to or above 35.5°C. However, contrary to our predictions, we did not find evidence for a relationship between associative learning performance, measured under either normal or high temperatures, and the antipredator response. This suggests that physiological constraints due to thermoregulation, rather than temperature-mediated learning impairment, are the primary drivers of variation in the antipredator response under high temperatures.

Our measure of antipredator response combined time spent vigilant, alarming and flying. While vigilance is unlikely to be particularly energetically costly because it only requires subtle head movements and is compatible with reduced activity (e.g. [60]), flying increases endogenous heat production [61] and sustained alarm calling may be energetically costly [62,63]. Accordingly, our post hoc analyses showed that the proportion of time spent vigilant was higher in response to the genet than the box, regardless of temperature; in contrast, individuals flew more when faced with the genet than the box under normal but not high temperatures (see electronic supplementary material, section S6). Although we did not find a significant temperature effect on the proportion of time spent alarming (see electronic supplementary material, section S6), the few individuals that engaged in active mobbing (repeated alarming for 8 s or more) all did so when presented with the genet under normal temperatures. Together, these results suggest that the reduced antipredator response under high temperatures was primarily due to a decrease in active behaviours, rather than a failure to detect the predator, supporting the hypothesis that physiological constraints from overheating shape antipredator behaviour. This aligns with recent studies in wild birds that found a lower likelihood of fleeing in several species of shorebirds [7] and a reduced mobbing response, which includes both alarm calling and flight, in great tits [8] under high temperatures. Additionally, in line with our findings, a previous study showed that wild Western Australian magpies did not decrease their vigilance in the heat; instead, they were more vigilant when presented with a potentially threatening stimulus (aeroplane noise) in the heat [60]. Staying vigilant without reacting actively by fleeing or alarming might be adaptive if predators are also less active in high temperatures.

Contrary to our expectations, we found no relationship between individual cognitive performance and antipredator response. One possible explanation is that the pied babblers did not recognize the taxidermied genet as a genuine predator threat. However, we found evidence that individuals responded more strongly to the genet compared to the control box under normal temperatures, and we recorded repeated alarming—a costly behaviour typically used in mobbing [10]—exclusively in response to the genet. In some cases, other bird species (fork-tailed drongos (*Dicrurus adsimilis*) and white-browed sparrow-weavers (*Plocepasser mahali*)) also joined in mobbing the model predator following the alarm calls of the focal bird. Collectively, these observations indicate that the genet was recognized as a predator.

Another possible explanation is that cognitive traits other than associative learning are more directly linked to behaviour during encounters with known predators. These could include long-term memory of predator cues [64], attention [65] and other stimulus valuation processes that may alter risk-sensitivity thresholds during decision-making in predator encounters [66]. Interestingly, studies in humans and captive animals have found that heat stress can impair both attention and long-term memory (reviewed in [22]). If the same holds true for wild birds, this could partly explain their reduced antipredator response in high temperatures. Future experiments could present associative learning tasks using different colour combinations under normal temperatures until the associations are learned and then present the same colour combinations later (e.g. 60 and 90 days) under either normal or high temperatures to test whether long-term memory of learned associations is reduced in the heat and whether impaired long-term memory is linked to reduced antipredator responses. Experiments could also investigate effects of impaired attention. For example, reaction time to a model predator could be measured under normal and high temperatures, with additional distractors (e.g. neutral objects or sounds that increase attentional load) present or absent. As a control, the same distractors could also be presented under both temperature conditions without a predator, allowing researchers to test whether attention declines in the heat. Alternatively, potential effects of impaired decision-making could be tested using ambiguous predator cues, such as partially hidden predator models. In parallel, to confirm that high temperatures impair decision-making *per se*, birds could first be trained to associate two artificially generated sounds (A and C) with an alarm call or a neutral conspecific call under normal temperatures, followed by presentation of intermediate B sounds under both normal and high temperatures to measure responses to ambiguous cues.

Finally, while impaired associative learning performance may not influence the response to known predators, it may affect the ability to learn about novel predators [19]. Future experiments could expose individuals to a novel predator alongside conspecific alarm calls under normal and high temperatures, then later assess their antipredator response under normal temperatures to determine whether heat-mediated learning impairment prevents recognition of the novel predator. Ultimately, understanding how heat-mediated cognitive impairment translates into altered behaviour is a key component for predicting how wild animal populations will adjust to global warming.

Overall, we provide novel empirical evidence for a reduced antipredator response under high temperatures in the wild, which could have implications for survival rates [4], as well as wildlife welfare [67]. In pied babblers, there is already evidence that, above 38°C, faecal glucocorticoid metabolites increase proportionally to air temperatures, indicating an acute stress response [68]. Predator threats typically trigger the sympathetic-adrenomedullary axis and may lead to a further rise in glucocorticoid levels via the hypothalamic–pituitary–adrenal axis, increasing stress [69]. Additionally, exposure to predation risk will be longer if thermoregulatory constraints prevent immediate escape. Therefore, heat stress and predator-induced stress may have a synergistic negative impact on individual welfare.

At the population level, predictive models integrating temperature thresholds for behavioural changes (e.g. resting versus active) with microclimate data and physiological parameters indicate that pied babblers will experience a steep increase in the risk of lethal hyperthermia by 2100 [70]. Additionally, population viability models based on current information on temperature-dependent survival and fecundity rates in the study population predict local extinctions by 2050 [30]. A reduced ability to respond to predators in the heat, as described here, could further worsen current predictions for the persistence of this and other species under global warming.

## Data Availability

Data and code are available on Figshare [71]. Supplementary material is available online [72].

## References

[1] IPCC. 2023 Summary for policymakers. In Climate change 2023: synthesis report. Contribution of working groups I, II and III to the sixth assessment report of the intergovernmental panel on climate change (eds HL Core Writing Team, J Romero), pp. 1–34. Geneva, Switzerland: IPCC.

[2] Cunningham SJ, Gardner JL, Martin RO. 2021 Opportunity costs and the response of birds and mammals to climate warming. Front. Ecol. Environ. **19**, 300–307. (10.1002/fee.2324)

[3] White RH *et al*. 2023 The unprecedented Pacific Northwest heatwave of June 2021. Nat. Commun. **14**, 727. (10.1038/s41467-023-36289-3) 36759624 PMC9910268

[4] Maille A, Schradin C. 2016 Survival is linked with reaction time and spatial memory in African striped mice. Biol. Lett. **12**, 20160346. (10.1098/rsbl.2016.0346) 27484646 PMC5014029

[5] Corregidor-Castro A, Militti S, Morinay J, Romano A, Morganti M, Cecere JG, Rubolini D, Pilastro A. 2025 Facing the heat: nestlings of a cavity-nesting raptor trade safety for food when exposed to high nest temperatures. Anim. Behav. **219**, 123006. (10.1016/j.anbehav.2024.10.020)

[6] Green BR, Tanner EP, Chandler RB, Abernathy HN, Conner LM, Garrison EP, Shindle DB, Miller KV, Cherry MJ. 2025 Temperature influences resource selection and predation risk tolerance in a climate generalist. Landsc. Ecol. **40**, 1–15. (10.1007/s10980-025-02056-6)40270488

[7] Gutiérrez JS, Catry T, Espinosa‐Colín M, Masero JA, Granadeiro JP. 2023 Heat stress increases risk taking in foraging shorebirds. Funct. Ecol. **37**, 1005–1019. (10.1111/1365-2435.14288)

[8] Cordonnier M, Ridley AR, Lengagne T, Dutour M. 2023 The impact of high temperatures on bird responses to alarm calls. Behav. Ecol. Sociobiol. **77**, 82. (10.1007/s00265-023-03354-2)

[9] Shettleworth SJ. 2009 Cognition, evolution, and behavior. New York, NY: Oxford university press.

[10] Carlson NV, Griesser M. 2022 Mobbing in animals: a thorough review and proposed future directions. Adv. Study Behav. **54**, 1–41. (10.1016/bs.asb.2022.01.003)

[11] Fišer O, Strnadová I, Veselý P, Syrová M, Němec M, Kamišová B, Šalom J, Fuchs R. 2025 Strange features are no better than no features: predator recognition by untrained birds. Anim. Cogn. **28**, 5. (10.1007/s10071-024-01924-z) 39775088 PMC11706896

[12] Wheeler BC, Fahy M, Tiddi B. 2019 Experimental evidence for heterospecific alarm signal recognition via associative learning in wild capuchin monkeys. Anim. Cogn. **22**, 687–695. (10.1007/s10071-019-01264-3) 31069567 PMC6687673

[13] Veen T, Richardson DS, Blaakmeer K, Komdeur J. 2000 Experimental evidence for innate predator recognition in the Seychelles warbler. Proc. R. Soc. Lond. B Biol. Sci. **267**, 2253–2258. (10.1098/rspb.2000.1276) PMC1690806 11413640

[14] Kacelnik A, El Mouden C. 2013 Triumphs and trials of the risk paradigm. Anim. Behav. **86**, 1117–1129. (10.1016/j.anbehav.2013.09.034)

[15] Kacelnik A, Bateson M. 1997 Risk-sensitivity: crossroads for theories of decision-making. Trends Cogn. Sci. **1**, 304–309. (10.1016/s1364-6613(97)01093-0) 21223933

[16] Dukas R. 2004 Evolutionary biology of animal cognition. Annu. Rev. Ecol. Evol. Syst. **35**, 347–374. (10.1146/annurev.ecolsys.35.112202.130152)

[17] Stahlschmidt ZR, Joura H, Makarem JR, Sun JL. 2024 Hot and scared: how do heatwaves and predation risk impact resource acquisition and allocation? Biol. Lett. **20**, 20240009. (10.1098/rsbl.2024.0009) 38653332 PMC11040502

[18] Stephens DW. 2008 Decision ecology: foraging and the ecology of animal decision making. Cogn. Affect. Behav. Neurosci. **8**, 475–484. (10.3758/cabn.8.4.475) 19033242

[19] Morand-Ferron J. 2017 Why learn? The adaptive value of associative learning in wild populations. Curr. Opin. Behav. Sci. **16**, 73–79. (10.1016/j.cobeha.2017.03.008)

[20] Wooster EIF, Gaynor KM, Carthey AJR, Wallach AD, Stanton LA, Ramp D, Lundgren EJ. 2024 Animal cognition and culture mediate predator–prey interactions. Trends Ecol. Evol. **39**, 52–64. (10.1016/j.tree.2023.09.012) 37839906

[21] Schmit C, Hausswirth C, Le Meur Y, Duffield R. 2017 Cognitive functioning and heat strain: performance responses and protective strategies. Sports Med. **47**, 1289–1302. (10.1007/s40279-016-0657-z) 27988874

[22] Soravia C, Ashton BJ, Thornton A, Ridley AR. 2021 The impacts of heat stress on animal cognition: implications for adaptation to a changing climate. Wiley Interdiscip. Rev. **12**, e713. (10.1002/wcc.713)

[23] Van Hook MJ. 2020 Temperature effects on synaptic transmission and neuronal function in the visual thalamus. PLoS One **15**, e0232451. (10.1371/journal.pone.0232451) 32353050 PMC7192487

[24] Gaoua N. 2010 Cognitive function in hot environments: a question of methodology. Scand. J. Med. Sci. Sports **20**, 60–70. (10.1111/j.1600-0838.2010.01210.x) 21029192

[25] Chang CH, Bernard TE, Logan J. 2017 Effects of heat stress on risk perceptions and risk taking. Appl. Ergon. **62**, 150–157. (10.1016/j.apergo.2017.02.018) 28411725

[26] Syndicus M, Wiese BS, van Treeck C. 2018 In the heat and noise of the moment: effects on risky decision making. Environ. Behav. **50**, 3–27. (10.1177/0013916516680700)

[27] Park RJ, Behrer AP, Goodman J. 2021 Learning is inhibited by heat exposure, both internationally and within the United States. Nat. Hum. Behav. **5**, 19–27. (10.1038/s41562-020-00959-9) 33020588

[28] Blackburn G, Broom E, Ashton BJ, Thornton A, Ridley AR. 2022 Heat stress inhibits cognitive performance in wild Western Australian magpies, Cracticus tibicen dorsalis. Anim. Behav. **188**, 1–11. (10.1016/j.anbehav.2022.03.016)

[29] Soravia C, Ashton BJ, Thornton A, Ridley AR. 2023 High temperatures are associated with reduced cognitive performance in wild southern pied babblers. Proc. R. Soc. B **290**, 20231077. (10.1098/rspb.2023.1077) PMC10688443 37989242

[30] Ridley AR, Wiley EM, Bourne AR, Cunningham SJ, Nelson-Flower MJ. 2021 Understanding the potential impact of climate change on the behavior and demography of social species: the pied babbler (Turdoides bicolor) as a case study. In Advances in the study of behavior (eds M Naguib, L Barrett, SD Healy, J Podos, LW Simmons, M Zuk), pp. 225–266. Cambridge, MA: Elsevier. (10.1016/bs.asb.2021.03.005)

[31] du Plessis KL, Martin RO, Hockey PAR, Cunningham SJ, Ridley AR. 2012 The costs of keeping cool in a warming world: implications of high temperatures for foraging, thermoregulation and body condition of an arid‐zone bird. Glob. Chang. Biol. **18**, 3063–3070. (10.1111/j.1365-2486.2012.02778.x) 28741828

[32] Jones KA, Krebs JR, Whittingham MJ. 2007 Vigilance in the third dimension: head movement not scan duration varies in response to different predator models. Anim. Behav. **74**, 1181–1187. (10.1016/j.anbehav.2006.09.029)

[33] Sealey BA, James LS, Cohen G, Ryan MJ, Page RA. 2024 Rapid foraging risk assessments in the Jamaican fruit bat, Artibeus jamaicensis. Anim. Behav. **216**, 45–53. (10.1016/j.anbehav.2024.07.015)

[34] Raihani NJ, Ridley AR. 2008 Experimental evidence for teaching in wild pied babblers. Anim. Behav. **75**, 3–11. (10.1016/j.anbehav.2007.07.024)

[35] Raihani NJ, Ridley AR. 2007 Adult vocalizations during provisioning: offspring response and postfledging benefits in wild pied babblers. Anim. Behav. **74**, 1303–1309. (10.1016/j.anbehav.2007.02.025)

[36] Bourne AR, Cunningham SJ, Spottiswoode CN, Ridley AR. 2020 Hot droughts compromise interannual survival across all group sizes in a cooperatively breeding bird. Ecol. Lett. **23**, 1776–1788. (10.1111/ele.13604) 32945068

[37] Humphries DJ, Nelson‐Flower MJ, Bell MBV, Finch FM, Ridley AR. 2021 Kinship, dear enemies, and costly combat: the effects of relatedness on territorial overlap and aggression in a cooperative breeder. Ecol. Evol. **11**, 17031–17042. (10.1002/ece3.8342) 34938490 PMC8668771

[38] Bourne AR, Cunningham SJ, Nupen LJ, McKechnie AE, Ridley AR. 2022 No sex‐specific differences in the influence of high air temperatures during early development on nestling mass and fledgling survival in the Southern Pied Babbler (Turdoides bicolor). Ibis **164**, 304–312. (10.1111/ibi.12990)

[39] Golabek KA, Radford AN. 2013 Chorus-call classification in the southern pied babbler: multiple call types given in overlapping contexts. Behaviour **150**, 691–712. (10.1163/1568539x-00003074)

[40] Raihani NJ, Nelson‐Flower MJ, Golabek KA, Ridley AR. 2010 Routes to breeding in cooperatively breeding pied babblers Turdoides bicolor. J. Avian Biol. **41**, 681–686. (10.1111/j.1600-048x.2010.05211.x)

[41] Wiley EM, Ridley AR. 2018 The benefits of pair bond tenure in the cooperatively breeding pied babbler (Turdoides bicolor). Ecol. Evol. **8**, 7178–7185. (10.1002/ece3.4243) 30073076 PMC6065330

[42] Mitchell D, Snelling EP, Hetem RS, Maloney SK, Strauss WM, Fuller A. 2018 Revisiting concepts of thermal physiology: predicting responses of mammals to climate change. J. Anim. Ecol. **87**, 956–973. (10.1111/1365-2656.12818) 29479693

[43] Thapa H. 2025 Effects of background risk on associative learning of zebrafish (Danio rerio). Doctoral Thesis, [Saskatoon, Canada]: University of Saskatchewan.

[44] Ashton BJ, Ridley AR, Edwards EK, Thornton A. 2018 Cognitive performance is linked to group size and affects fitness in Australian magpies. Nature **554**, 364–367. (10.1038/nature25503) 29414945 PMC5815499

[45] Shaw RC, Boogert NJ, Clayton NS, Burns KC. 2015 Wild psychometrics: evidence for ‘general’ cognitive performance in wild New Zealand robins, Petroica longipes. Anim. Behav. **109**, 101–111. (10.1016/j.anbehav.2015.08.001)

[46] Rowe C, Healy SD. 2014 Measuring variation in cognition. Behav. Ecol. **25**, 1287–1292. (10.1093/beheco/aru090)

[47] Hollén LI, Bell MBV, Wade HM, Rose R, Russell A, Niven F, Ridley AR, Radford AN. 2011 Ecological conditions influence sentinel decisions. Anim. Behav. **82**, 1435–1441. (10.1016/j.anbehav.2011.09.028)

[48] Dutour M, Kasper J, Ridley AR. 2021 Transfer of information between a highly social species and heterospecific community members. Behav. Ecol. Sociobiol. **75**, 1–11. (10.1007/s00265-021-03075-4)

[49] Ghalambor CK, Martin TE. 2002 Comparative manipulation of predation risk in incubating birds reveals variability in the plasticity of responses. Behav. Ecol. **13**, 101–108. (10.1093/beheco/13.1.101)

[50] Peter A. 2017 Solomon coder, beta version 17.03.22. See https://solomoncoder.com/.

[51] Caro TM. 2005 Antipredator defenses in birds and mammals. Chicago, IL: University of Chicago Press.

[52] R.C. Team. 2024 R: A Language and Environment for Statistical Computing. Vienna, Austria: R foundation for statistical computing.

[53] Brooks ME, Kristensen K, Van Benthem KJ, Magnusson A, Berg CW, Nielsen A, Skaug HJ, Mächler M, Bolker BM. 2017 glmmTMB balances speed and flexibility among packages for zero-inflated generalized linear mixed modeling. R J. **9**, 378. (10.32614/RJ-2017-066)

[54] Adams DB, Kitchen DM. 2020 Model vs. playback experiments: the impact of sensory mode on predator‐specific escape responses in saki monkeys. Ethology **126**, 563–575. (10.1111/eth.13008)

[55] Bilby J, Colombelli-Négrel D, Katsis AC, Kleindorfer S. 2022 When aggressiveness could be too risky: linking personality traits and predator response in superb fairy-wrens. PeerJ **10**, e14011. (10.7717/peerj.14011) 36193436 PMC9526405

[56] Lê S, Josse J, Husson F. 2008 FactoMineR: an R package for multivariate analysis. J. Stat. Softw. **25**, 1–18. (10.18637/jss.v025.i01)

[57] Symonds MRE, Moussalli A. 2011 A brief guide to model selection, multimodel inference and model averaging in behavioural ecology using Akaike’s information criterion. Behav. Ecol. Sociobiol. **65**, 13–21. (10.1007/s00265-010-1037-6)

[58] Hartig F. 2022 DHARMa: Residual Diagnostics for Hierarchical (Multi-Level / Mixed) Regression Models. R package version 0.4.6. See https://CRAN.R-project.org/package=DHARMa.

[59] Gray L, Webster MM. 2023 False alarms and information transmission in grouping animals. Biol. Rev. **98**, 833–848. (10.1111/brv.12932) 36653332

[60] Blackburn G, Ashton BJ, Ridley AR. 2024 Evidence that multiple anthropogenic stressors cumulatively affect foraging and vigilance in an urban-living bird. Anim. Behav. **211**, 1–12. (10.1016/j.anbehav.2024.02.014)

[61] Nudds RL, Bryant DM. 2000 The energetic cost of short flights in birds. J. Exp. Biol. **203**, 1561–1572. (10.1242/jeb.203.10.1561) 10769218

[62] Luther D, Danner R. 2016 Males with larger bills sing at higher rates in a hot and dry environment. Auk **133**, 770–778. (10.1642/auk-16-6.1)

[63] Stoddard PK, Salazar VL. 2011 Energetic cost of communication. J. Exp. Biol. **214**, 200–205. (10.1242/jeb.047910) 21177941 PMC3008630

[64] de Faria CM, de Souza Sá F, Lovenstain Costa DD, da Silva MM, da Silva BC, Young RJ, de Azevedo CS. 2020 Captive-born collared peccaries learning about their predators: lessons learnt but not remembered. Behav. Process. **171**, 104031. (10.1016/j.beproc.2019.104031) 31899275

[65] Dukas R. 2002 Behavioural and ecological consequences of limited attention. Phil. Trans. R. Soc. Lond. B Biol. Sci. **357**, 1539–1547. (10.1098/rstb.2002.1063) 12495511 PMC1693070

[66] Leavell BC, Bernal XE. 2019 The cognitive ecology of stimulus ambiguity: a predator–prey perspective. Trends Ecol. Evol. **34**, 1048–1060. (10.1016/j.tree.2019.07.004) 31416642

[67] Beaulieu M. 2024 Capturing wild animal welfare: a physiological perspective. Biol. Rev. **99**, 1–22. (10.1111/brv.13009) 37635128

[68] Moagi LL, Bourne AR, Cunningham SJ, Jansen R, Ngcamphalala CA, Ganswindt A, Ridley AR, McKechnie AE. 2021 Hot days are associated with short-term adrenocortical responses in a southern African arid-zone passerine bird. J. Exp. Biol. **224**, b242535. (10.1242/jeb.242535) 34032270

[69] Creel S. 2018 The control of risk hypothesis: reactive vs. proactive antipredator responses and stress‐mediated vs. food‐mediated costs of response. Ecol. Lett. **21**, 947–956. (10.1111/ele.12975) 29744982

[70] Conradie SR, Wolf BO, Cunningham SJ, Bourne A, van de Ven T, Ridley AR, McKechnie AE. 2024 Integrating fine‐scale behaviour and microclimate data into biophysical models highlights the risk of lethal hyperthermia and dehydration. Ecography **2025**, e07432. (10.1111/ecog.07432)

[71] Camilla S, Benjamin JA, Sara PM, Alex T, Amanda RR. 2025 Datasets analysed in the manuscript titled ‘Investigating the relationship between heat-mediated cognitive impairment and antipredator response in a wild bird’. Figshare. (10.6084/m9.figshare.28938089) PMC12503953 41063729

[72] Soravia C, Ashton BJ, Piquet-Morón S, Thornton A, Ridley AR. 2025 Supplementary material from: Investigating the relationship between heat-mediated cognitive impairment and antipredator response in a wild bird. Figshare. (10.6084/m9.figshare.c.8022508) PMC12503953 41063729

